# Evaluating Adherence of Health-Care Team to Standard Guideline of Colistin Use at Intensive Care Units of a Referral Hospital in Shiraz, Southwest of Iran

**DOI:** 10.15171/apb.2017.047

**Published:** 2017-09-25

**Authors:** Afsaneh Vazin, Iman Karimzadeh, Atiyeh Zand, Nazafarin Hatami-Mazinani, Dena Firouzabadi

**Affiliations:** Department of Clinical Pharmacy, Faculty of Pharmacy, Shiraz University of Medical Sciences, Shiraz, Iran.

**Keywords:** Colistin guideline, Adherence, Clinical Pharmacist, Intensive care unit

## Abstract

***Purpose:*** To evaluate colistin use according to global standard drug consumption in intensive care units of a referral hospital in Shiraz, Iran

***Methods:*** A prospective, interventional study was performed during an 11 month period on 100 patients admitted to ICUs of a teaching hospital being treated with colistin for at least 3 subsequent doses. Required demographic, clinical, and paraclinical data were gathered by a pharmacist. Fifteen indexes were considered to evaluate colistin use. A clinical pharmacist reviewed indication and dose of colistin at the time of prescribing this agent.

***Results:*** In our study population, pneumonia (69%) was the main indication of colistin. In 87% of patients, colistin administration was based on microbiological laboratory evidence. Continuation of therapy was inappropriate in 5% of cases. By the intervention of the clinical pharmacist, colistin was discontinued in all patients in whom empirical therapy was continued incorrectly. None of the patients received loading dose of colistin. The maintenance dose, dose interval, and duration of treatment of colistin were appropriate in 76%, 71%, and 100% of patients, respectively. For none of the patients, the pharmacokinetic dosing method was used. In all patients, serum creatinine and WBC count were evaluated on daily basis. The sum indexes of colistin use were relevant to standard guidelines in 67.33% of the cases.

***Conclusion:*** The results of this study highlight the necessity of the pharmaceutical care team participation in all stages of treatment with antibiotics. After pharmacist interventions, some criteria of colistin utilization were corrected and brought closer to standard values.

## Introduction

*Acinetobacter* is a non-fermentative gram negative coccobacilli with high capability for colonization in human body.^1^* Acinetobacter baumannii* is an invasive and resistant microorganism existing in immunosuppressed patients such as Intensive Care Unit (ICU) patients on mechanical ventilation.^2^* A. baumannii* infection has become a clinical concern due to its ability to produce wide spectrum antibiotic resistance, either intrinsic or acquired.^3^

Colistin was derived from a microorganism named *Bacillus polymyxa*, a Colistinus subspecies. It was first used to treat patients infected with gram negative microorganisms in 1959.^4-6^ The most important adverse effects of colistin, when given intravenously, are renal and neurological toxicities.^7^ In some patients, allergic reactions, gastrointestinal events, and pulmonary toxicities have been also reported.^8^ Later, by the introduction of more available and less toxic antibiotics such as aminoglycosides, the rate of colistin usage was decreased. Unfortunately, by the increase in the prevalence of multiple drug-resistant (MDR) hospital-acquired infections, use of colistin has been risen once again.^9^

Adverse effects, drug resistance, the outburst of MDR organisms, and serious pressure on limited medication costs are the major complications of antibiotic misuse.^10,11^ Drug use evaluations (DUE) are administrative approaches to both improve the pattern of use and reduce the cost of medications. Programs evaluating the use of drugs are structural, innovative and progressive in which they determine a drug’s pattern of consumption in different situations and diseases and its goal is to reduce inappropriate medication administration, enhancing medication effectiveness, and reducing medication costs.^12,13^

To the best of our knowledge, no study has been done on the DUE of colistin so far. For the first time, we decided to evaluate colistin use pattern according to global standards in the ICUs of a referral hospital in Iran. Beside that, interventions of a clinical pharmacist in detecting and correcting inappropriate dose and indication of colistin in the same settings were also studied.

## Materials and Methods

### Study setting

A prospective, interventional study was performed during an 11 month period from December 2014 to September 2015 on patients admitted to 4 internal, general, central, and emergency ICUs of Nemazee hospital, a general multispecialty, referral, tertiary health-care setting, affiliated to Shiraz University of Medical Sciences, Shiraz, Iran. The Institutional Review Board and the Medical Ethics Committee of the hospital approved the study.

### Study population

All patients being treated with colistin that received at least 3 subsequent doses of the antibiotic were included in this study. Patients received less than three doses of colistin, due to any reason such as ward transfer, hospital discharge, or death were excluded from the study.

### Data collection

Data gathering was done by a general pharmacist under the supervision of a senior clinical pharmacy attending. Demographic data including age, sex, height, and total body weight were collected and the patient’s ideal body weight was calculated accordingly. The patient’s medical history, diagnosis, reason for patient’s referral to the ward, final diagnosis, pre-existing medical conditions, and whether the patient’s infection was acquired from community or hospital, were all recorded. Laboratory data consisting microbiological culture, hematologic parameters including white blood cell (WBC), biochemical data including blood urea nitrogen (BUN), serum creatinine, and immunologic factors (procalcitonin, c-reactive protein [CRP]) were also recorded before and during the course of colistin treatment. To evaluate colistin use compared to available guidelines and recommendations, fifteen indexes were taken into account. Each index was scored as either 0 or 1.

These items were as follow: 1) indication, 2) preparation and dilution, 3) administration route, 4) duration of each infusion, 5) administration of loading dose, 6) daily dose, 7) administration intervals, 8) duration of treatment according to isolated pathogen, 9) evaluation of the microbiological culture before prescription, 10) evaluation of microbiological culture 48-72 hours after administration, 11) considering the patient’s BUN and creatinine before prescribing, 12) monitoring serum creatinine level periodically during the treatment, 13) reducing the dose or discontinuation in patients who developed colistin nephrotoxicity (defined by a rise in serum creatinine of more than 0.3 mg/dl within 48 hours or a 1.5 folds increase compared to baseline values within 7 days, or a decrease in urine volume to less than 0.5 ml/kg/h for more than 6 hours), 14) re-evaluation of dosing if needed (in cases of change in serum creatinine or partial clinical response), and 15) the presence of a moderate or major interaction between colistin and other co-administered medications identified by the Lexi-Interact online version. In addition to the above indexes, the patients were also monitored for probable adverse effects of colistin.^14^

Concurrent with the screening phase of the study, the drug and therapeutic committee of the hospital was committed a clinical pharmacist to review and verify colistin orders regarding indication and dose in the studied ICUs at the time of initiating this agent. In the case of unnecessary indication or inappropriate dose, the clinical pharmacist gave his corrective suggestions.

### Statistical analyses

Continuous and categorical variables were expressed as mean ± standard deviation (SD) and percentage, respectively. Descriptive analyses were carried out by the Statistical Package for the Social Sciences (SPSS) version 20 software (IBM company, New York, NY, United States).

## Results

During the study period, a total of 126 patients were primarily screened. Among them, 100 were eligible to be included in the study. Forty three percent, 21%, 19%, 17% of the study populations were admitted to the internal, general, central, and emergency ICUs, respectively. The causes of patient drop out from the study were as follows: death (71%), transfer to other wards (23%), and early discontinuation of colistin (11%). Demographic data of patients have been demonstrated in Table 1. Their mean ± SD age was 56.70 ± 18.18 years. Sixty four percent of the cohort was male.

**Table 1:** Demographic and clinical characteristics of the study population (n = 100)

**Demographic and clinical data**		**Value**
Age (year)	Mean ± SD	56.70 ± 18.18
	Range	22-96
	Sex, Male/Female ratio	64/36
Weight (Kg)	Mean ± SD	68.05 ± 12.61
	Range	45-95
Height (Cm)	Mean ± SD	167.36 ± 8.76
	Range	150-190
Ideal body weight (Kg)	Mean ± SD	62.4 ± 9.78
	Range	45.5-84
Infection, n (%)	Pneumonia	69 (69)
	Sepsis	14 (14)
	Wound infection	9 (9)
	Urinary tract infection	5(5)
	Pericarditis	3 (3)
Infection type, n ‏(%)	Community acquired	3 (3)
	Hospital acquired	97 (97)

Colistin indication for each patient has been listed in Table 2. Ventilator associated pneumonia (69%) was the most common indication of colistin followed by sepsis (14%), and skin/soft tissue infection (9%). Regarding source of infection, 97% were categorized as hospital acquired. Colistin treatment in 87% cases was based on the microbiological culture data (definite therapy) and the remaining (13%) were classified as an empiric. Considering the involved organism, most (78%) cases were *Acinetobacter* spp. followed by *Klebsiella* spp. (16%) and *Pseudomonas* spp. or *Escherichia coli* (6%).

**Table 2:** Colistin indications in the study population (n = 100)

**Indication**	**N (%)**
Pneumonia	69 (69)
Sepsis	14 (14)
Skin/soft tissue infection	9 (9)
Urinary tract infection	5 (5)
Peritonitis	3 (3)

Colistin was administered as an intravenous infusion in all patients. Its route of administration was correct in 100% of patients. In 39% of the cases, the methods of preparation and dilution were correct. Duration of infusion was correct in 65% of patients and in the remaining 35%, it was less than 30 minutes. The mean ± SD duration of colistin therapy was 15.61 ± 11.55 days. Treatment duration of colistin ranged between 2 and 21 days. The administration time interval and duration of treatment were correct in 71% and 84% of patients, respectively.

The pharmacokinetic dosing method was used for no patients. Seventy six percent of the administered doses at the beginning of the treatment were appropriate. Among inappropriate initial doses, 22% and 2% were lower and higher than those recommended, respectively. Loading dose was not administered for any of the patients during the study period. Colistin maintenance dose was appropriate in 76% of cases during the treatment course. In 13% of patients in whom colistin was started empirically, continuation of therapy was inappropriate in 5% of cases. Colistin was discontinued in all these patients after the clinical pharmacist’s intervention. Among cases of definite therapy, the isolated pathogen was sensitive to other antibiotics rather than colistin (7%). In most of the patients (94%), no data regarding microbiological culture had been provided 48-72 hours after initiating therapy with colistin. The acceptance rates of physicians about clinical pharmacist interventions regarding colistin indication and dose were 100% and 89%, respectively.

In all patients, WBC count was done daily from the first to last day of colistin treatment. At the first day of colistin administration, WBC count of 57% of the study population was more than 10,000/μl. In contrast, only 31% of the cohort had WBC counts above 10,000/μl at the end of colistin treatment. Microbiological culture and decision making accordingly were done for 61% of the patients. In more than half (54%) and one-third (36%) of the cohort, CRP and body temperature were considered as indexes of colistin treatment response. Other factors such as serum procalcitonin were also taken into account in 9% of the cases.

In all patients, serum creatinine was evaluated on daily basis. In 91% of the patients, the first dose of colistin was adjusted according to the patient’s serum creatinine and BUN. In 45% of the patients who developed nephrotoxicity, no action was taken; However in the remaining 55%, the time intervals of colistin administration were increased. For 39% of the patients, re-evaluation of colistin dose had to be made according to relevant serum creatinine. But no dose adjustment had been done for 51% of these patients. According to the fact that our study population was all critically ill patients with poor conditions and on the other hand, they were received sedative medications, evaluating the neurologic toxic effects of colistin was not feasible. Allergic reactions to colistin were not seen in any patient.

In 29% of the patients, a level D potential drug interaction (according to Lexi-Interact definition) with colistin was identified. These drug-drug interactions have been brought in Table 3. No drug interaction level X was identified.

**Table 3:** Type, probable mechanism, severity, and frequency of moderate and major potential drug interactions of colistin with co-administered medications in the study population (n = 100)

**Drug-drug interaction**	**Probable mechanism**	**Severity**	**Percentage**
Colistin + Vancomycin	Vancomycin may enhance the nephrotoxic effects of colistin	Moderate	51.7
Colistin + Amphotericin B	Amphotericin B may enhance the nephrotoxic effects of colistin	Moderate	31
Colistin + Aminoglycosides	Aminoglycosides may enhance the nephrotoxic effects of colistin	Moderate	24.1
Colistin + Atracurium	Atracurium may enhance the neurotoxic effects of colistin	Moderate	3.4

Table 4 has summarized the 15 studied indexes of colistin use in our cohort. The mean ± SD score of colistin use indexes in the study population was 10.1 ±1.72. In other words, the sum indexes of colistin use were in accordance to standard guidelines in 67.33% of the cases.

**Table 4:** Fifteen indexes of colistin use in the study population (n = 100)

**No**	**Index**	**Appropriate/Performed, percent**	**Inappropriate/Not performed, percent**
1	Indication	88	12
2	Method of preparation and dilution	39	61
3	Administration route	100	0
4	Duration of each infusion	65	35
5	Administration of a loading dose	0	100
6	Maintenance daily dose	76	24
7	Administration time interval	71	29
8	Duration of treatment	84	16
9	Evaluation of the microbiological culture before prescription	87	13
10	Evaluation of microbiological culture 48-72 hours after administration	6	94
11	Considering the patient’s renal function before prescribing	91	9
12	Monitoring serum creatinine level periodically during the treatment	100	0
13	Reducing the dose or discontinuation in patients who developed colistin nephrotoxicity	55	45
14	Re-evaluation of dosing if needed	39	51
15	Considering moderate or major interaction between colistin and other co-administered	29	71

## Discussion

Due to the fact that antibiotic resistance has become an important issue worldwide and awareness regarding incorrect and excessive antibiotic use in hospitals has become more, most antibiotic stewardship programs have aimed towards limiting the use of these medications. Limiting the use of certain groups of antibiotics can help to achieve this goal.^15^ Results of a study in the United States showed that limiting the use of antibiotics has been considered in 56% of the newly published guidelines and also in 81% of medical teaching centers.^9^ On the other hand, decreasing hospital acquired infections can lead to less antibiotic use such as colistin, and also reduction in the rate of resistant or MDR microorganisms.

In this study, we evaluated colistin use pattern in 4 ICUs of a referral hospital in Shiraz. Considering the fact that a high percentage of infections originate from hospitals, it is mandatory to implement better policies to prevent hospital acquired infections. The rate of carbapenem resistant *A. baumannii* isolates in Shiraz hospitals is concerning that necessitates taking special measures.^16^

Low rates of colistin-resistant *Acinetobacter* spp. have been reported in Iran.^17^ For instance, 1% of *Acinetobacter* spp. isolates were resistant to colistin at Hamedan in 2011-2012, 11.6% at Isfahan in 2011-2012, and 12% at Tehran in 2009-2010. However, more recent studies from other parts of Iran like Tehran, Ahvaz, and Kermanshah have demonstrated that the susceptibility of *Acinetobacter* spp. to colistin has been 100%.^18-22^ Similar data have been reported from at least two surveys in Nemazee hospital within 2008-2009 and 2013.^16,23^ Therefore, colistin in combination with other appropriate antibiotics can be considered as the last antibacterial

drug in the treatment of MDR infections caused by gram negative pathogens in our center.

In our study population, pneumonia was the main indication (about 69% of the cases) of colistin. In 87% of patients, colistin administration was based on microbiological evidence. In contrast, colistin was prescribed empirically in only 13% of the cases. This issue can be justified partially by exploiting carbapenem discs for determining the antimicrobial susceptibilities of *Acinetobacter spp.* according to the last Clinical and Laboratory Standard Institute (CLSI) guideline. This led physicians to choose other antibiotics rather than colistin as their first choice. This action was started from the winter of 2014 in the microbiological laboratory of Nemazee hospital.

By the intervention of pharmaceutical care unit, colistin was discontinued in all patients in whom empirical therapy was continued incorrectly. In a prospective cohort study done by Reina *et al.* on colistin in 2005, 185 patients admitted at the ICU for more than 48 hours and had either *Acinetobacter spp*. or *Pseudomonas aeruginosa* infections, were included. The population was divided into two groups and for 55 of them, colistin and for the remaining 130 individuals, other antibiotics were prescribed. It was demonstrated that inappropriate empirical therapy with colistin was much more common than that with other antibiotics (100% versus 8%, respectively). Interestingly, the cure rate on the 6^th^ day of admission and also death rate were similar in both groups.^24^

A number of studies have demonstrated that physicians have a tendency towards empirical prescription of broad spectrum antibiotics. However, some of these empirical prescriptions have been for the treatment of serious and life-threatening infections. Also in patients with poor condition, use of a great number of broad spectrum antibiotics for a prolonged duration has been implicated.^25^ On the other hand, in 7% of our patients that microbiological culture had been done before starting treatment, colistin was prescribed inappropriately and the culture results indicated that patients were sensitive to more available and less toxic antibiotics. In all such cases, colistin was replaced with other appropriate antibiotics such as carbapenems after the clinical pharmacist intervention.

Similar to other antibacterial agents, obtaining a second microbiological culture 48 to 72 hours after the start of colistin therapy has been recommended to evaluate patient response to the treatment. This was done for only 6% of our patients. However, noting that ventilator associated pneumonia (69%) was the most common indication of colistin in our cohort. Obtaining a second microbiological culture 48 to 72 hours after the start of colistin therapy is crucial for evaluating response to treatment in only certain infections such as sepsis.

In our study, colistin was administered only as an intravenous infusion. Duration of colistin infusion has been stated to be at least 30 minutes according to manufacturer package insert. In 65% of our study population, this duration was correct. Reconstitution and dilution of colistin was not done based on reference standards in 61% of cases. Therefore, the concentration of the resulting solution was lower than that was mentioned elsewhere. This medication is commonly available as two parenteral formulations worldwide. European and US products have expressed doses as sodium colisthimetate and colistin base, respectively. Eighty milligram of sodium colisthimetate is almost equivalent to 30 mg of colistin base activity.^26^ According to the drug information provided by the United States manufacturers, the recommended maximum daily dose is 10,000,000 IU (800 mg) based on sodium colistimetate that is about two times the maximum dose recommended by the European manufacturers (6,000,000 IU or 480 mg). Unfortunately, most physicians are not aware of such differences and usually use data provided by the United States that recommends dosing according to colistin base. This can lead to the prescription of either higher or lower doses of colistin required and each can lead to toxicity or failure in treatment of the patient, respectively.

Routine monitoring of either serum or plasma concentration of colistin has not been well established yet; however according to drug information provided by the manufacturer, especially in cases in whom treatment failure or toxicity is probable, specifically in neonates and patients with cystic fibrosis, therapeutic drug monitoring (TDM) can be beneficial. Colistin serum concentration was not determined in any of our study population. Since there is the possibility of delayed action of colistin, microbial resistance, treatment failure and rise in mortality, a loading dose of 9,000,000-12,000,000 IU has been suggested.^27^ Nevertheless, none of our patients was given the loading dose of colistin. This may be partially due to lack of knowledge of physicians regarding the possibility of administering the loading dose. Another reason might be their concern over developing colistin nephrotoxicity. Lack of sufficient medication available in the Iranian pharmaceutical market can be another reason for this finding in our study.

During the study, nephrotoxicity developed in 31% of the patients. Different definitions have been used for determining colistin nephrotoxicity, but in recent studies as ours, RIFLE criteria have been mostly used.^27,28^ In this study, we found out that about two-thirds of the patients that developed nephrotoxicity while being treated with colistin also received other nephrotoxic agents, mainly vancomycin. Incidence of colistin nephrotoxicity was reported to be ranged from zero to 54%. These variations can be due to different criteria used for the definition of nephrotoxicity, different study populations, different clinical status of patients, and the presence of risk factors for nephrotoxicity such as co-administration of nephrotoxic agents.^26^

It has been recommended that serum creatinine should be evaluated regularly during colistin treatment. Similarly, it was done for our cohort. It seems that in the current study setting, serum creatinine was evaluated daily as a routine practice of the wards for all patients received colistin without considering its changing trend. In some cases, colistin nephrotoxicity may appear even after the discontinuation of therapy, but it seems unlikely that this would be the reason for daily serum creatinine monitoring after stopping colistin.

The time interval of colistin administration was increased in about 55% of the patients who developed nephrotoxicity. According to treatment protocols, in the case of rise in patient’s serum creatinine during therapy, either dose adjustment or discontinuation of therapy can be considered. Colistin pharmacokinetics has inter-individual and intra-individual variations and therefore requires individualized dosing. Considering this issue, colistin dose adjustment was needed for 39% of our patients. However, it was not performed for 51% of them. This may be due to fact that no attention was paid by the clinicians to dosing concepts according to pharmacokinetic of colistin.

In prescribing antibiotics, different factors such as the selection of the correct dose, correct administration, and the interval between doses are important and some issues such as obtaining culture before antibiotic administration, adverse drug events, TDM, laboratory data, the correct time to give the antibiotic and duration of therapy are other substantial factors that have to be considered.^15^ In our cohort, the sum indexes of colistin use were relevant to standard guidelines in 67.33% of the cases. However, this score is not the actual as well as comprehensive index of colistin utilization in our population because scoring all the mentioned indexes for the whole patients was not feasible. In summary, the most important weak points in administering colistin in our cohort were as follows: not administering loading dose, incorrect method of reconstitution and dilution, and lack of dose adjustment in certain conditions. In contrast, positive points of colistin utilization in the study population were the correct route of administration and duration of infusion, serum creatinine monitoring on a daily basis, appropriate indications, obtaining microbiological data before starting therapy, and correct dose as well as interval of administration. Because of relatively small sample size, statistical analyses were not possible and selection of larger sample size can lead to better and more reliable results. The acceptance rate of clinical pharmacist’s intervention by physicians was 100% regarding colistin indication. However, regarding dose and time interval of administration with the acceptance rate of 89%, less attention was paid to the clinical pharmacist’s comments. This could be due to lack of clinical pharmacist’s follow up after the intervention. Therefore, the persistent and regular attendance of pharmacists in daily rounds of the ward can reduce medication errors in the pharmacotherapy processes. Interestingly, the acceptance rate of clinical pharmacist interventions in the current survey was within the range reported from both European (73-89%) and American (85-99%) studies.^29^

## Conclusion

Our data demonstrated that different indexes of colistin use were in accordance to standard guidelines in 67.33% of the cases. In addition, after clinical pharmacist interventions, some criteria of colistin utilization regarding dose and indication were corrected and brought closer to standard values. In health-care settings, not enough attention is usually paid to pharmacokinetics of the medication and also its preparation and dilution and also in our case, ignoring loading dose administration especially in critically ill patients. Active and reciprocal collaboration of pharmacists with physicians can help towards improving pattern of use and can hopefully motivate the healthcare group in establishing better policies for antibiotic use.

## Acknowledgments

This research was performed by Atieh Zand in partial fulfillment of the requirements for certification as a pharmacist. The present paper was adopted from the proposal number 93-01-05-8461 approved by vice-chancellor for research affairs of Shiraz University of Medical Sciences.

## Ethical Issues

The Institutional Review Board and the Medical Ethics Committee of the hospital approved the study.

## Conflict of Interest

The authors declare no conflict of interests.
